# Comparative studies on thin polycaprolactone-tricalcium phosphate composite scaffolds and its interaction with mesenchymal stem cells

**DOI:** 10.1186/s40824-018-0153-7

**Published:** 2019-01-03

**Authors:** Gopinathan Janarthanan, In Gul Kim, Eun-Jae Chung, Insup Noh

**Affiliations:** 1 Department of Chemical and Biomolecular Engineering, Seoul, 01811 Republic of Korea; 2 Convergence Institute of Biomedical Engineering and Biomaterials, Seoul National University of Science and Technology, Seoul, 01811 Republic of Korea; 3 Department of Otorhinolaryngology-Head and Neck Surgery, Seoul National University Hospital, Seoul, Republic of Korea

**Keywords:** Hybrid, Poly(ε-caprolactone), Tricalcium phosphate, Gelatin, Thin films

## Abstract

**Background:**

Hybrid scaffolds combining biodegradable polymers and ceramic particles for control of cell adhesion and proliferation are interesting materials for tissue engineering applications. Combinations of biodegradable polymers and ceramics are to provide higher beneficial functionalities to tissue engineering scaffolds with addition of different cell specific bio-factors. Many such hybrid combinations have been reported by several researchers around the world by using various methods and solvents as well as bioactive matrix polymers to fabricate such biomaterials. However, thin hybrid scaffolds with high porosity, cell adhesion factors and biodegradability, as well as the ability to support stem cells often require tedious processes like electrospinning, freeze drying, etc. A simple method to develop porous biodegradable hybrid scaffolds with proper cell adhesion factors is still the need of the hour in tissue engineering and regenerative medicine.

**Method:**

Thin biodegradable poly(ε-caprolactone) (PCL) based hybrid scaffolds were developed in combination with α-tricalcium phosphate (TCP) particles, gelatin and fibronectin separately and the fabricated scaffolds were evaluated systematically using human mesenchymal stem cells (hMSCs) for tissue engineering applications. A simple modified solvent casting method combined with gas foaming process was used to develop porous thin hybrid structures and compared their properties with those of corresponding non-porous hybrid scaffolds. The TCP particles distribution, morphology, biodegradability and functional groups of the different hybrid scaffolds were analyzed using energy-dispersive X-ray spectroscopy (EDX), light microscopy/scanning electron microscopy (SEM), buffer solutions and Fourier-transform infrared spectroscopy (FTIR), respectively The cellular and tissue regeneration behaviors such as in vitro cell attachment (live/dead assay), cell proliferation (CCK-8 assay) and histological studies were performed using hMSCs.

**Results:**

Thin PCL-based hybrid scaffolds were fabricated using modified solvent casting method. Homogeneous distribution of TCP particles in the scaffolds were confirmed by EDX. Cellular interactions of the hybrid scaffolds demonstrated overall higher cell adhesion, proliferation and tissue regeneration on the non-porous thin films of PCL-TCP, PCL-TCP-gelatin and PCL-TCP-fibronectin. Coating of fibronectin was remarkable in induction of cell adhesion and proliferation.

**Conclusions:**

The experimental results revealed that diversely designed PCL-TCP thin hybrid films showed high cell interaction and proliferation with hMSCs. From the results of the cell viability, attachment, proliferation and histological analyses as well as their biodegradation and coating effects, we conclude that these thin PCL-TCP hybrid films are suitable for tissue engineering applications.

## Background

Scaffolds for bone tissue engineering aim to repair or regenerate bone defects by working as a platform for controlling biological functions of bone cells [1–5]. An ideal scaffold for tissue engineering should have similar mechanical and biochemical properties as the native tissues. Since polymer or ceramic itself does not meet the requirements of biomaterials for their tissue engineering applications, hybrid scaffolds such as biodegradable polymer/calcium phosphate hybrid scaffolds are promising biomaterials for bone tissue engineering. As examples, the pure polymeric biomaterials demonstrated lower mechanical properties than that of natural bone, and the inorganic materials based scaffolds made of are too brittle to be handled [6–8]. Hybridization of calcium phosphate with polymers, however, has been considered as a popular method to overcome their disadvantage properties as fabricate hybrid scaffolds. Calcium phosphate as bioactive and osteo-conductive in hybrid scaffolds has been reported to not only improve the geometry, topography and mechanical behaviors of polymeric scaffolds, but also enhance bone cell adhesion and induce the differentiation of osteo-progenitor cells [9–11].

A variety of forms of hybrid scaffolds were recently reported such as manufacturing porous hybrid materials, coating a hybrid scaffold with a bioactive polymeric layer [12], and a nano-fibrous scaffold by electrostatic co-spinning [13, 14]. Two approaches of incorporation of inorganic materials have been reported such as mixing inorganic materials with polymer directly [15, 16] and depositing inorganic materials on the surface of polymeric scaffolds such as electrospinning methods [17, 18]. Mixing the inorganic materials with polymer could improve the mechanical property, but, it is difficult to optimize the surface of the scaffold to maintain cell compatibility because of the encapsulation of inorganic materials. Compared to mixing method, depositing mineral or bioactive materials on the surface of polymer matrix have possibility of not only enhancing mechanical properties but also providing a favorable surface for cell attachment and proliferation in tissue engineering applications [19–21]. As an example, Ruhe et al. [21] reported recombinant human bone morphogenetic protein 2 (rhBMP-2) release behavior from rhBMP-2 loaded poly(lactide-*co*-glycolide)-calcium phosphate cement. They reported that rhBMP-2 release depended on their hybrid composition and nanostructure, as well as the pH of the experimental medium.

Even though there are many advantages of hybrid scaffolds, control of interaction between inorganic and polymeric biomaterials is another big challenge, because of the absence of strong interactions between these two interfaces. Furthermore, incorporation of bioactive molecules potentially deteriorates mechanical properties of the hybrid scaffolds. Ratier et al. [22] reported a decrease in compressive strength with an increase in antibiotic tetracycline concentration. Another issue of the hybrid scaffolds is not strong cell adhesion on their surface, leading to difficulty in enhancement of tissue engineering. Polymeric biomaterials such as gelatin, chitosan and alginate have been also employed to improve the resistance-to-washout and handling properties of calcium phosphates since these biomaterials disintegrate on early exposure with blood and other biological fluid [23]. Furthermore, coating of cell-adhesive polymers such as fibronectin [24, 25] and collagen [26, 27] among many extracellular matrix polymers has been reported to increase cell adhesion and proliferation in their applications to tissue engineering including biodegradable poly(ε-carprolactone) (PCL) scaffold [28, 29].

In this study, biodegradable PCL and α-tricalcium phosphate (TCP) particles were selected as the hybrid biomaterials, whereas gelatin and fibronectin as cell adhesion factors. Considering the different aspects of hybrid scaffolds, we fabricated a hybrid film in a thin film form and then tested them by fabricating different composition and forms of PCL, PCL-TCP, PCL-gelatin and fibronectin-coated PCL scaffolds with and without micro-pores for tissue engineering applications. All the developed thin films were compared and analyzed for biocompatibility and effects of porosity and morphological shapes using mesenchymal stem cells(MSCs) cells in vitro and the results are promising to be applied in tissue engineering applications.

## Methods

### Materials

Poly(ε-caprolactone) (PCL) pellets (Mw: 80000–90,000) and gelatin (Type B, bovine) were purchased from Sigma Aldrich (St. Luis, MO, USA). The solvent 2,2,2-trifluoroethanol (TFE) and 1,1,1,3,3,3-hexafluoro-2-propanol (HFP) were procured from Alfa Aesar (Korea). Fibronectin, ammonium bicarbonate, tricalcium phosphate were purchased from Sigma Aldrich Chemical Co. (St. Luis, MO, USA, Germany and China). Tissue culture reagents such as fetal bovine serum (FBS, Biotechnics Research, Mission Viejo, CA, USA), penicillin-streptomycin (Lonza; Seoul, Korea), 0.05% trypsin- ethylene diamine tetra-acetic acid (EDTA)-1X (Gibco-Life Technologies; Carlsbad, California, USA), and live & dead viability/cytotoxicity kit for mammalian cells (Invitrogen, Carlsbad, CA, USA) were purchased and used. Mesenchymal stem cells (Seoul National University Hospital, Seoul, Korea) were used for biocompatibility tests and distilled water (DW) was employed for all the experiments.

### Synthesis

#### Synthesis of alpha-tricalcium phosphate (α-TCP)

α-tricalcium phosphate micro-particles was synthesized by following the protocol reported in our previous paper [4]. Briefly, after mixing calcium hydrogen phosphate dihydrate (68.84 g) with calcium carbonate (20.02 g) in a molar ratio of 2:1, 100 mL ethanol was added. The mixture was transferred into a 500 mL alumina pot (d = 5 mm) containing 100 g of zirconia particles. Afterwards, the 3 component mixtures were mixed with a ball milling machine (Model: SBML-2, SciLab Korea Co.; Seoul, Korea) with 150 rpm for 24 h. Then, the zirconia particles were retrieved by a sieve (Daehan Science; Seoul, Korea) with 425 μm pores. The mixture of dicalcium phosphate and calcium carbonate in powder was transferred to an aluminum tray and ethanol was removed by keeping it in a heating oven at 70 °C for 24 h. The powder in alumina pot was heated up in an electrical furnace (Model: MHS-160526-01, MiR Korea; Kwangju-city, Korea) at 1300 °C for 16 h using Segment-1 program (escalating of heating for 2 h and heating for 16 h and cooling by power off). Clusters of α–TCP particles were obtained after cooling. The α–TCP clumps were moved into an engineering plastic pot containing mixture of 2 different kinds of zirconia particles (300 g for d = 20 mm and 100 g for d = 4 mm) and 300 mL ethanol. After loading the plastic pot in the ball milling machine, the machine was operated by rotating at room temperature for 24 h. Finally, after collection of the dried α–TCP particles removing the ethanol at 70 °C in a heating oven, they were analyzed by a particle analyzer and used for fabrication of PCL hybrid films.

#### Preparation of thin porous and non-porous PCL hybrid films with α-TCP particles

Modified solvent casting method was used to prepare the non-porous and porous hybrid-polymeric composite scaffolds. For preparation of non-porous PCL-TCP scaffolds, PCL (10% *w*/*v*) was dissolved in 10 ml of TFE using magnetic stirrer at 300 rpm (50 °C) for 4 h. To this solution, α-TCP particles (10% *w*/w to PCL) were added and dispersed using a homogenizer at 500 rpm for 1 h and followed by sonication for 20 min. The composite solution was casted on to the Teflon evaporating dishes, air dried in fume hood for one day and followed by 3 days in vacuum oven at room temperature. Then, dried scaffolds were carefully immersed in diluted citric acid (5% w/v) for 5 min to remove the salt particles through gas foaming method. A gas foaming process was accomplished by drying under vacuum for one week, and then residual salts were eliminated by immersing it into an excess amount of warm water (40 °C). The porous hybrid PCL + TCP scaffolds were prepared similarly and ammonium bicarbonate (10% *w*/w to PCL) (~ 150–300 μm) particles were added and mixed well with PCL-TCP solution just before casting. For all samples, equal amounts of solutions were casted to maintain the thicknesses of the scaffolds. Control PCL porous films were prepared using the same method; however, PCL nonporous films were prepared without adding TCP or ammonium bicarbonate particles. All the prepared scaffolds were stored at 4 °C until further use.

#### Preparation of PCL hybrid films with gelatin incorporation

PCL (0.5 g) was dissolved in 5 ml of TFE and HFP by stirring overnight (300 rpm, room temperature). Under same conditions, gelatin (0.5 g) was separately dissolved in 5 ml of the similar solvent combination. Both PCL and gelatin solutions were mixed directly to yield a single solution with an equal weight ratio (1:1) of PCL and gelatin. This solvent combination evaporates faster than other solvents under normal conditions without affecting the film formation. The both porous and non-porous PCL-gelatin films were prepared by solvent casting. The solution was poured into teflon evaporating dishes and kept overnight under the fume hood to allow the TFE/HFP to evaporate for non-porous films. For porous PCL-gelatin films, sieved ammonium bicarbonate (~ 150–300 μm) were added into the solution before casting for porous films. Gas foaming process was carried out to fabricate the PCL-gelatin porous films and the films were rinsed mildly with distilled water for 5 min and the films were dried under vacuum for one week.

#### Fibronectin coating on PCL hybrid films

Coating of fibronectin (FN) on the hydrophobic surface of PCL-TCP particle hybrid film without gelatin were prepared by soaking the film in FN solution (5 mg/mL in distilled water) at 37 C for 1 h. The FN-coated films were then rinsed with distilled water. The dried scaffolds were stored until use. The scaffolds were sterilized prior to in vitro cell culture studies.

#### Morphological characterizations of hybrid films by digital camera, scanning electron microscope and energy-dispersive X-ray spectroscopy (EDX) analysis

After observation of hybrid film’s morphological images with digital camera, their morphological images of the PCL-TCP particle hybrid, their images were taken by light microscopy (Olympus, Japan). The morphological images of the hybrids with and with pores were also observed with SEM at different magnifications under inert environment after drying in − 78 C lyophilizer and then platinum coating for 1 min. The dry hybrid samples were fixed in advance on double sided tape on an aluminum stub. The distribution of TCP in the hybrid scaffolds were confirmed by analyzing the calcium distribution on the surface of the scaffolds using EDX analysis at different places of the sample.

### In vitro human mesenchymal stem cells (hMSCs) study

#### In vitro cell culture studies using hMSCs

Each sample was sterilized in ultraviolet light (UV) for 12 h. Human adipose-derived mesenchymal stem cells (hMSCs; STEMPRO®, R7788–110) were transplanted in growth medium (MesenPRO RSTM, Basal Medium / Growth supplement) for a certain period. Each of the prepared samples was transferred to non-adherent 12-well tissue culture plates and hMSCs were inoculated at a concentration of 2 × 10^4^ cells per scaffold. Seven sample groups were classified as follows; 1) Tissue culture plate (control) 2) PCL film, 3) PCL film coated with gelatin 4) PCL film with TCP particles 5) porous PCL films (gelatin coating), 7) porous PCL films with TCP particles (FN coating). The cell culture medium was changed every 2 days and proliferated for 5 days for cytotoxicity evaluation.

#### Cell proliferation assay

Cell counting (Cell Counting Kit-8, CCK-8; Dojindo, Biomax,; Seoul Korea) was performed on the 5th day after cell inoculation to confirm the cell proliferation rate. Each scaffold was transferred to a new 12-well plate and filled with 1 ml of fresh medium per well. Then, 100 μl of CCK-8 solution was added and incubated at 37 °C for 2 h. The reaction solution was transferred to a 96-well plate (100 μl) and the absorbance (OD) was measured at the wavelength of 450 nm using a microplate reader (Thermo Scientific).

#### Live/dead assay

Cell viability on all hybrid films was evaluated by the live and dead assay after in vitro mesenchymal stem cell culture for 5 days. Live and dead viability/cytotoxicity assay for mesenchymal stem cell was processed according to the protocol suggested by the vendor Invitrogen (Thermo Fisher Scientific, Seoul, Korea). PBS washing was twice performed, and then the assay solutions that was composed of 1.2 μL of 2 mM ethidium homodimer-1 and 0.3 μL of 4 mM calcein AM (dead and live stains, respectively) in 600 μL PBS. In vitro cell viability in gel was observed by a fluorescence microscope (Leica DMLB, Germany) after 30 min incubation in 37 °C in the 5% CO_2_ incubator.

#### Histological analyses

The PCL-TCP hybrid thin films containing hMSCs cells were stained with haematoxylin & eosin Y (H&E) and Masson’s trichrome (MT) stain to evaluate the formation of overall extracellular matrix and collagen and to observe the nucleus of the cells using the histological analysis. The hybrid films (1 cm × 1 cm) were sterilized and seeded with hMSCs cells (1 × 10^4^ cells) and cultured in standard conditions. At regular intervals (7 and 14 days), the samples were stained and imaged using inverted microscope. Staining procedure was performed according to our previous work [30].

#### Biodegradation test

##### Biodegradation test in simulated body fluid (SBF)

Six films of PCL, porous PCL film, PCL-gelatin film, porous PCL-gelatin films, PCL-TCP films and porous PCL-TCP films were cut into a dimension of 1 cm × 1 cm and weighed 20 mg each. Then 20 mg of the PCL pellet was measured and placed in 3 wells in a 24 well plate. Subsequently, 2 ml of SBF was added to each well, and then placed in a shaker at room temperature. The decomposition results are measured at different time intervals.

##### Biodegradation test in Dulbecco’s modified Eagle’s medium (DMEM)

All procedures were the same as using SBF. Instead of SBF, 2 ml of DMEM was added to each well and their weight was measured at the same interval.

### Statistical analysis

All data were represented as mean ± standard deviation. Statistical significance was tested with one-way and multi-way ANOVA by using the SPSS 18.0 program (ver. 18.0, SPSS Inc.; Chicago, IL, USA). The comparisons between two groups were evaluated by t-test, where the significant level was *p* < 0.05.

## Results

### Synthesis of thin PCL- TCP particles hybrid films

**Fig. 1:**
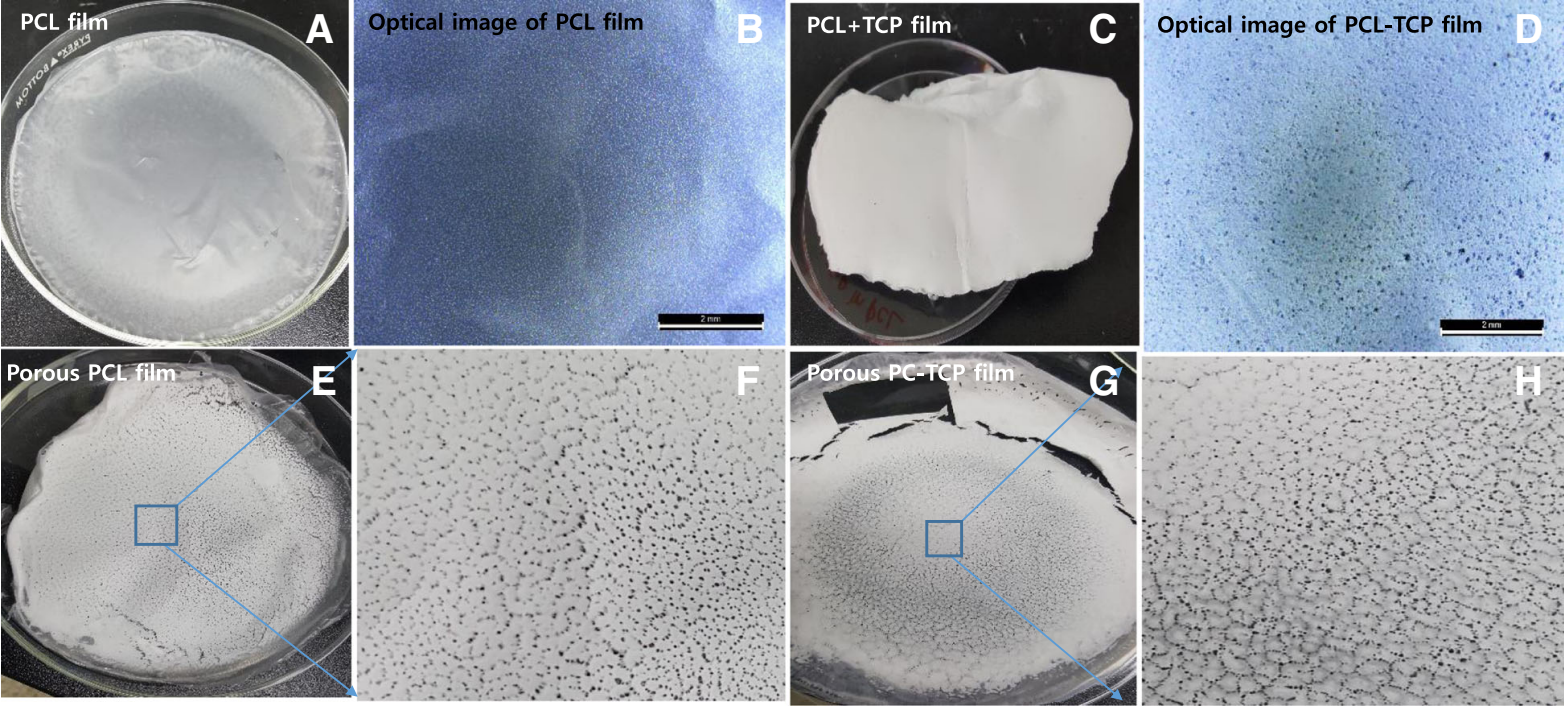
Films of PCL and PCL-TCP before and after pore generation observed by light microscope (**b**, **d**) and digital images (**a**, **c**, **e**, **f**, **g**, **h**)

We synthesized thin PCL films, PCL-TCP particles films, PCL-gelatin films and PCL-TCP particles-gelatin films. Porous films were obtained by gas forming of ammonium bicarbonate particle. While PCL films showed smooth surface (Fig. 1a, b), addition of TCP particles induce small pore shapes (Fig. 1c, d). Fabrications of porous films by porogen ammonium bicarbonate particles induced pores and crack-like shape (Fig. 1e, f, g, and h). In the porous forms, addition of TCP particles induced lager sizes of pores (Fig. 1g, h).

**Fig. 2:**
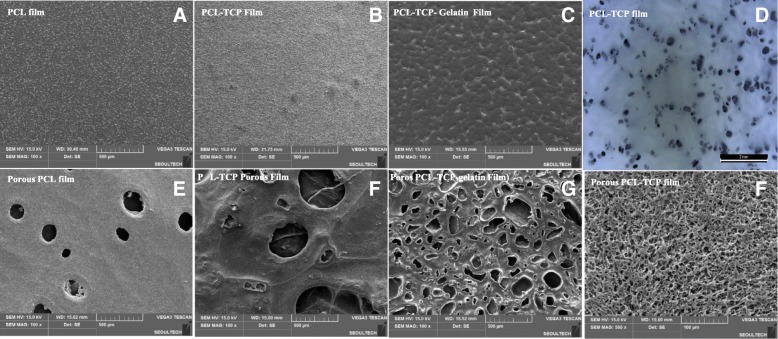
SEM analysis (100X) of PCL film **a** PCL-TCP particles hybrid either without **b** or with **c** gelatin inclusion; **d** PCL-TCP particles film, **e** Porous PCL film, porous PCL-TCP film, **f** Porous PCL-TCP-gelatin film, and high magnification (500X) of PCL-TCP film. D is image of light microscope

The porous films have pore sizes ranging approximately 100 to 200 μm in diameter (Fig. 2a, b, c, e, f, g) with smaller pores of approximately 10 μm as observed in SEM pictures (Fig. 2f). Light microscope observation demonstrated clearly distributions of TCP particles in PCL matrix (Fig. 2d). In specific, even the films consisted of no visible pores at low magnification, very small surface pores which were created as shown in the PCL-TCP particle film (Fig. 2d) because of solvent evaporation are seen under high magnification. The overall surface of the films was smooth without any large pores.

The presence of calcium phosphate particles (TCP) on the surface of the films were confirmed using EDX as demonstrated below. Homogeneous distribution of TCP particles can be confirmed from the image in Fig. 2d. The thicknesses of the films were observed in the range of 40 ± 6 μm. These films consisted of both large and small pores at low magnification, because of the usage of ammonium bicarbonate particles as well as mild leaching of gelatin. The pores were penetrating inside the films and not only on the surface of the films. The thicknesses of the porous films were in the range of 54 ± 6 μm.

### Chemical analysis

**Fig. 3:**
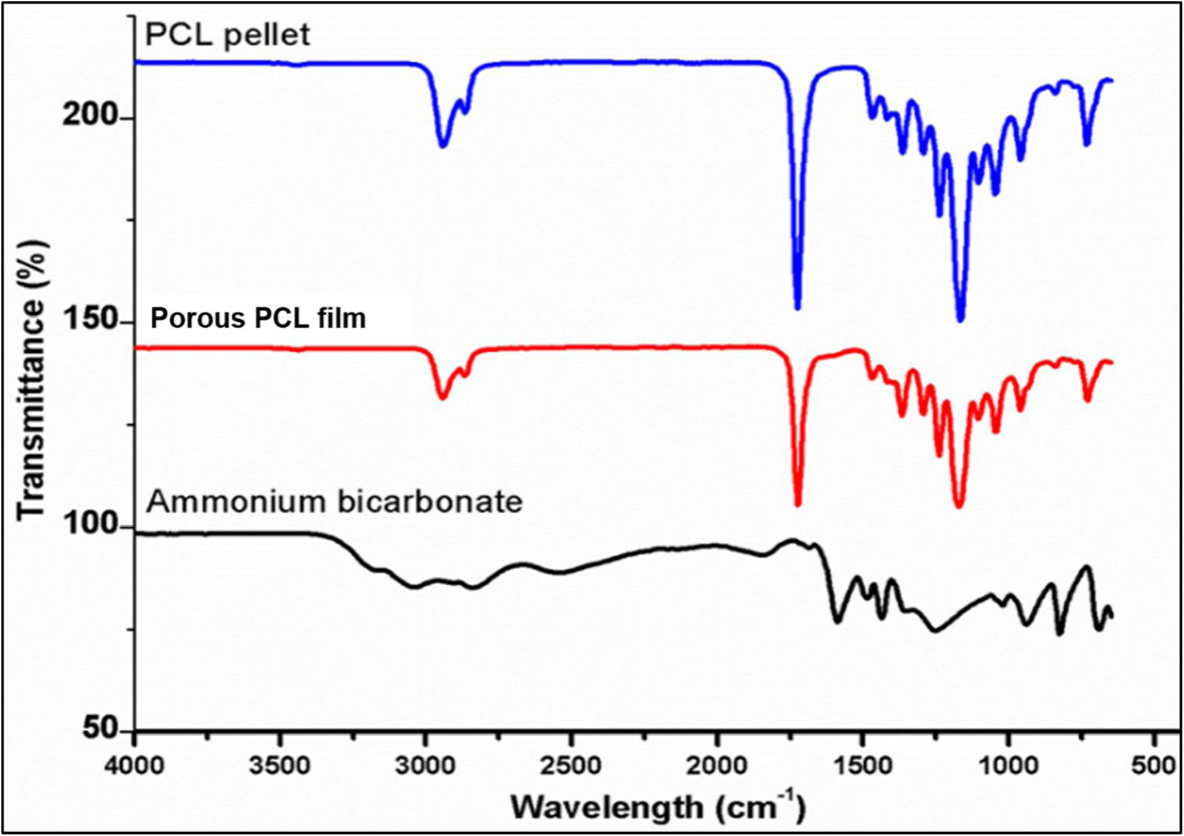
FTIR of porous PCL film compared with those of PCL pellets and ammonium bicarbonate particles

The chemical structures of porous PCL films were compared with PCL pellet and ammonium bicarbonate, in order to confirm the complete removal of ammonium bicarbonate particles from the films after gas foaming process. The FTIR spectrum of PCL pellets and porous PCL films showed the characteristic peaks of PCL and no ammonium bicarbonate peaks were observed in the porous PCL films (Fig. 3). The C-H (~ 2860 cm^− 1^) and C=O (~ 1720 cm^− 1^) stretching peaks correspond to the characteristic PCL were observed in the PCL pellet and PCL porous film spectrum (Fig. 3). These results were similar to the previous reports [31].

**Fig. 4:**
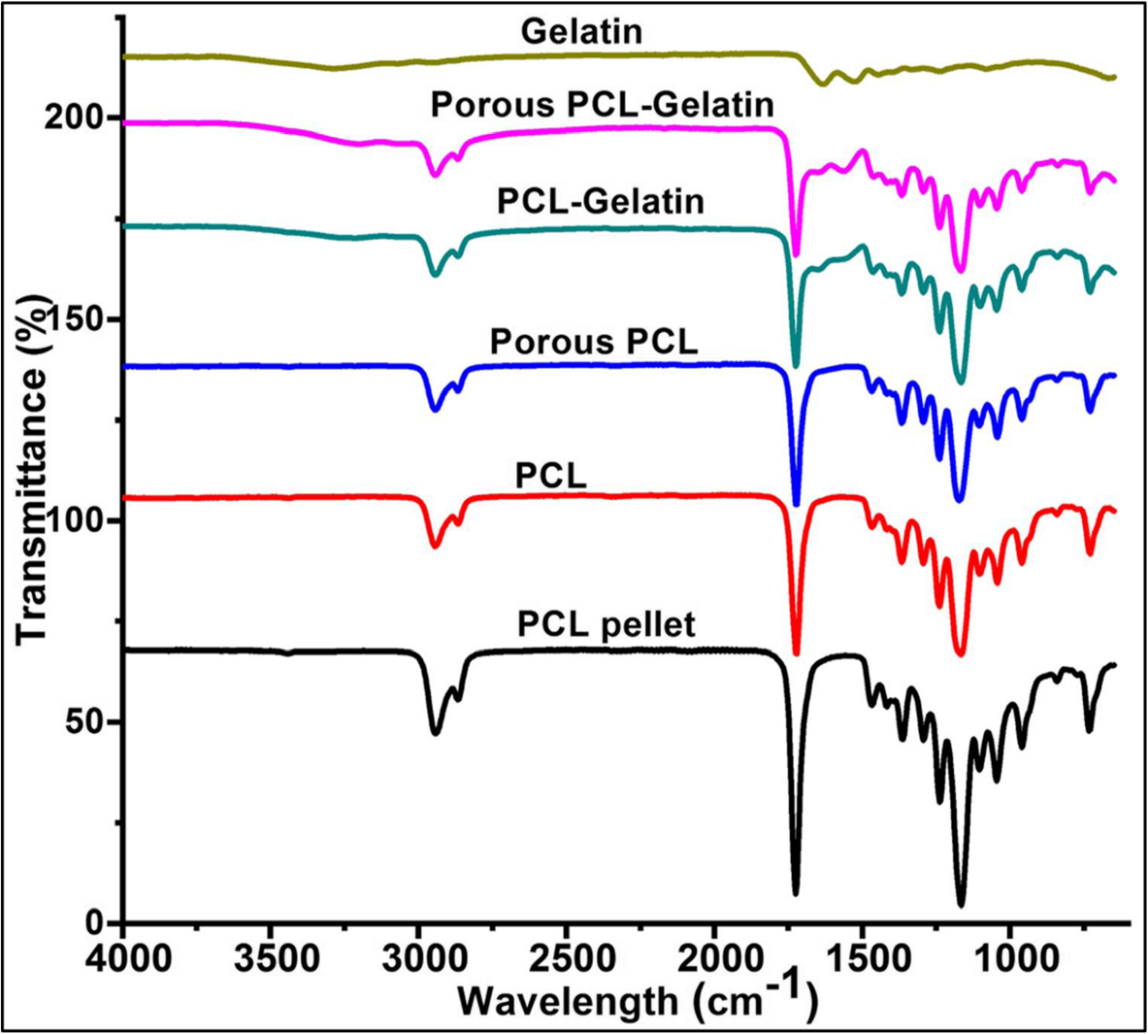
FTIR analysis of diverse PCL-TCP films

Next, we tried to detect gelatin in the PCL films and PCL-TCP films by FTIR. Figure 4 showed characteristic peaks of PCL polymers and new peaks of gelatin in the PCL- gelatin films. As described earlier, the characteristic peaks C-H (~ 2860 cm^− 1^) and C=O (~ 1720 cm^− 1^) of PCL are observed in all the PCL hybrid films, PCL film and PCL pellet.

The Fig. 4 shows the FTIR spectra of all the hybrid scaffolds. In PCL containing samples, the main characteristic peaks of PCL were observed at ~ 1727 cm^− 1^ (carbonyl stretch) and ~ 2949 cm^− 1^ (CH2 asymmetric stretch). Followed by the other peaks including symmetric CH_2_ stretching at ~ 2865 cm^− 1^, stretching of C-O and C-C at ~ 1293 cm^− 1^, asymmetric C-O-C at ~ 1240 cm^− 1^, symmetric C-O-C at 1170 cm^− 1^ were observed in all PCL containing films. [32] The characteristic peaks of gelatin include the peaks near ~ 3500–3250 cm^− 1^, ~ 1640 cm^− 1^ and 1550 cm^− 1^ mainly due to the effect of N-H bonds, C=O bonds and N-H bond wagging, respectively. These peaks are clearly visible in the gelatin spectrum. Also, the PCL-gelatin hybrid films also showed strong PCL characteristic peaks and mild gelatin peaks at these regions confirming the hybrid formation at related regions (PCL-gelatin and PCL-gelatin porous). However, these gelatin characteristic peaks are not seen in other PCL samples. [33, 34] In PCL-gelatin films, the characteristic peaks of both PCL and gelatin tend to appear as merged resulting from the possible interactions between the PCL’s ester group and the gelatin’s amine group.

**Fig. 5:**
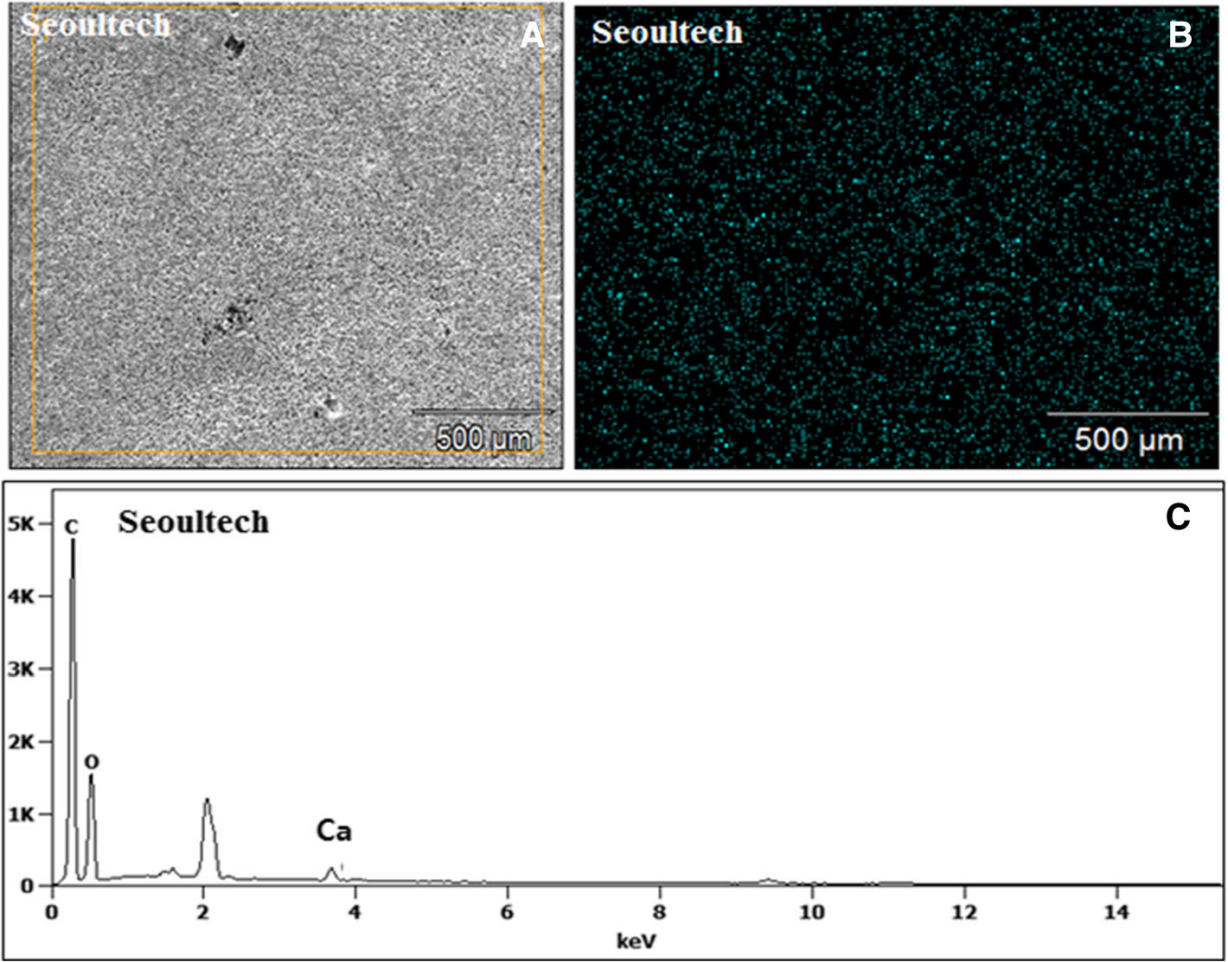
EDX of the PCL-TCP film, where **a** corresponds to the actual SEM image of the PCL-TCP film area where EDX was performed, and **b** represents the calcium mapping on the PCL-TCP film and **c** shows the different chemical composition of the PCL-TCP film

These films consisted of visible large pores at low magnification (Fig. 5a), because of the usage of ammonium bicarbonate particles. The overall surface of the films was smooth with large pores and the presence of calcium (from TCP) on the surface of the films was confirmed using EDX (Fig. 5b). Homogeneous distribution of TCP particles can be confirmed from this image (Fig. 5c). The thicknesses of the films were measured in the range of 40 ± 4 μm.

### Biodegradation in SBF and DMEM

**Fig. 6:**
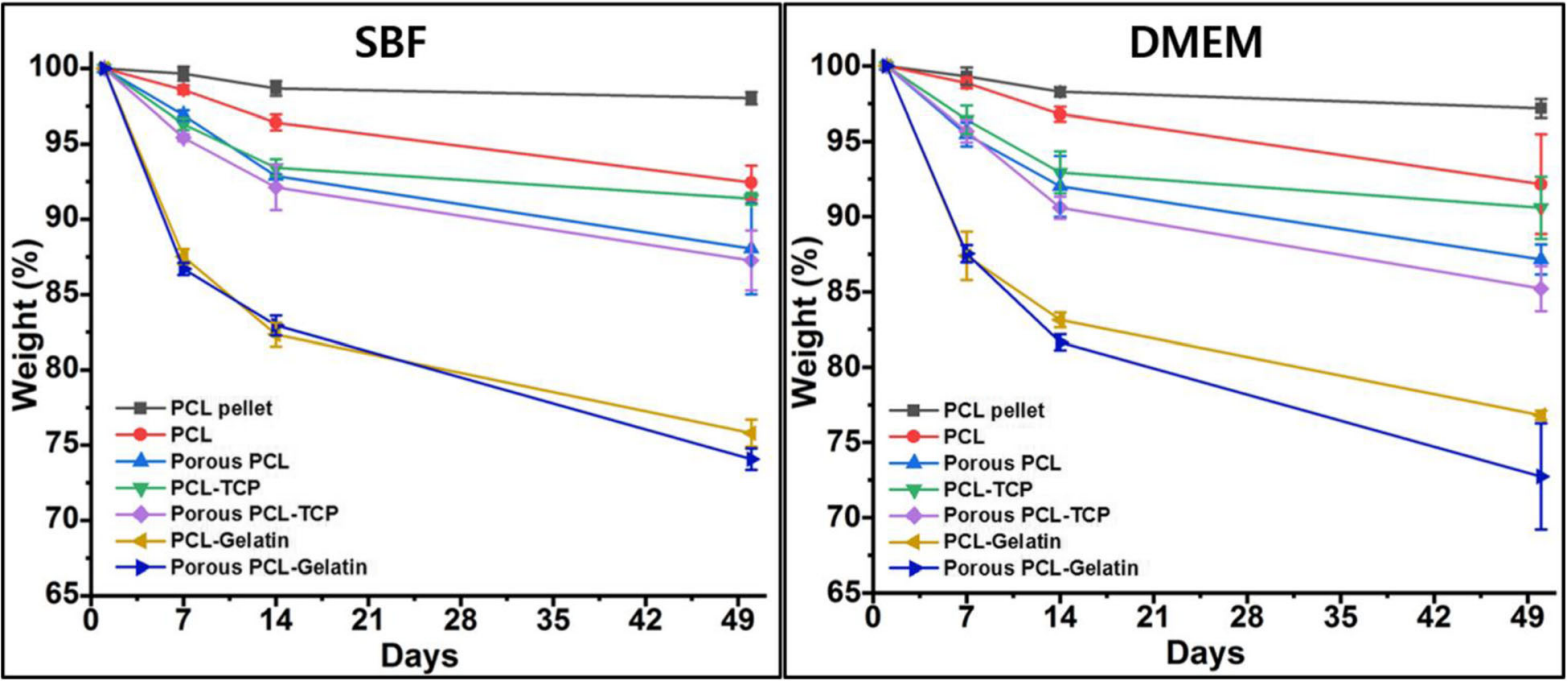
Biodegradation of PCL-TCP films in body simulated fluid and DMEM buffer

Figure 6 shows degradation of different hybrid films in SBF and DMEM up to 50 days. In both SBF and DMEM, the degradation trend was similar and however, there was slightly enhanced degradation percentage was observed in DMEM medium. Compared to the PCL pellets, all other films showed higher degradation till 50 days of the investigation. Porous samples showed relatively higher degradation compared to their non-porous films. This may be attributed to the availability of higher superficial surface area for hydrolysis in the porous samples. This trend was observed for all the porous samples irrespective of the sample composition. Among the PCL, PCL-TCP and PCL-gelatin samples, PCL-gelatin samples showed higher degradation rate. Generally, the PCL a degradable semi-crystalline synthetic polymer shows surface degradation initially when exposed to water. First the amorphous regions are targeted for hydrolysis followed by the crystalline regions [35]. Also, there is a possibility of random hydrolytic cleavages in PCL with high molecular weights as they are aliphatic polyesters in nature [36]. Due to this random breakage of polymer chains may lead to the release of the incorporated cell adhesion factors from the scaffolds by diffusion. This also depends on the homogeneous distribution of the such factors in the scaffolds [37]. The weight loss in hybrid scaffolds can be attributed to both polymer matrix degradation as well as the release of incorporated particles from the films. Hence, hybrid porous structures with cell adhesion factors show higher degradation in vitro; however, these scaffolds have the ability to match the loss by increased cell adhesion and proliferation on to the scaffolds.

### In vitro cell culture

**Fig. 7:**
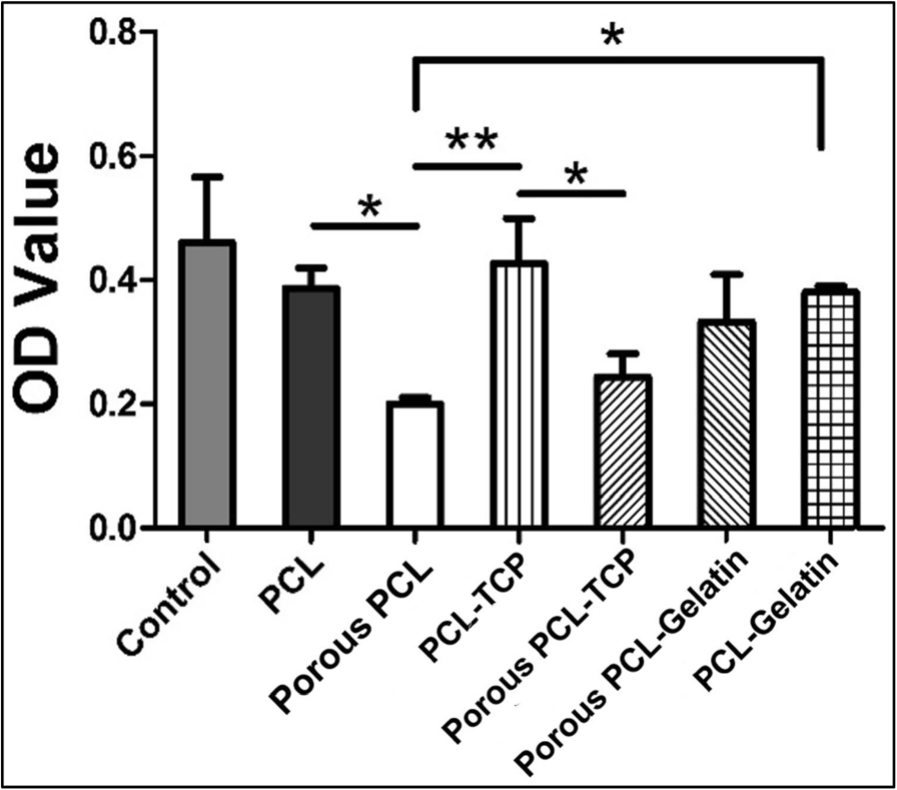
Proliferation of hMSCs on variously designed PCL-TCP film surfaces either with or without pore structures on days 5 via a CCK-8 assay

In vitro culture of mesenchymal stem cells was done on all these films such as PCL film, porous PCL film, PCL-TCP film and porous PCL-TCP film, PCL-gelatin film and porous PCL-gelatin film by comparing with that of control polystyrene culture flask (Fig. 7). Non-porous three groups (PCL, PCL-TCP, PCL-gelatin) show lower rate of cell proliferation rates than control. Normally inclusion of micro-particles like TCP particles induce changes in their surface morphologies, and gelatin has been known well as a cell-adhesive protein in tissue engineering. These results show that inclusion of TCP with morphological effects seemed to interfere little bit with cell proliferation. The group of porous PCL-gelatin films showed a slightly higher mean value than those of the other pore forming groups. However, the group with pores tended to have significantly lower OD value probably due to cell loss during seeding into the pores. All three experimental groups without pores showed no significant difference in cell numbers in statistics.

**Fig. 8:**
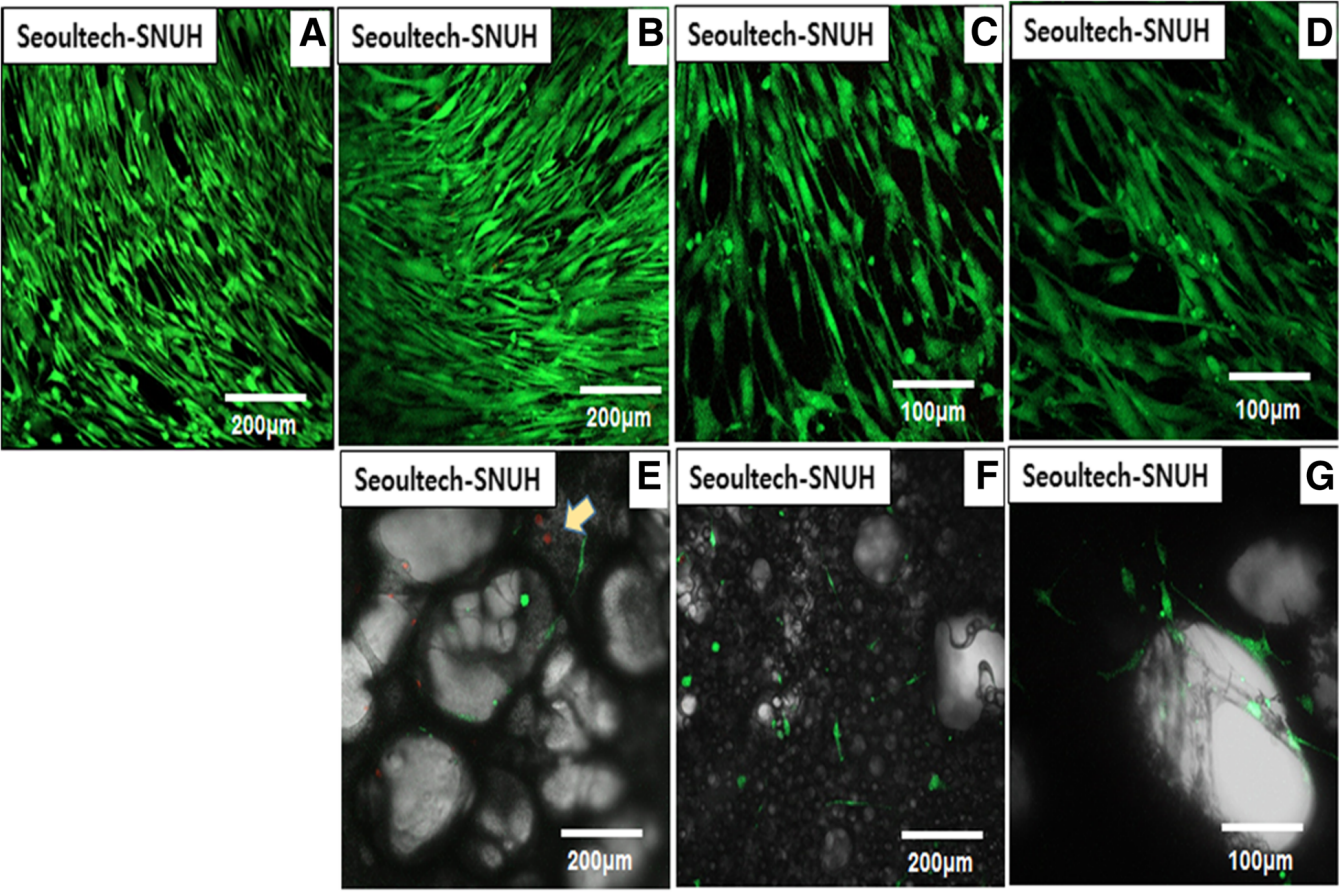
Cell proliferation and viability. Fluorescence micrographs of hMSCs grown on the surfaces of various PCL + TCP film with or without pore structure. Live cells are stained green, while dead cells are stained red. **a** Control (Tissue culture plate), **b** PCL film (FN coating), **c** PCL-gelatin film, **d** PCL-TCP film (FN coating), **e** porous PCL film (FN coating), **f** porous PCL-gelatin film, **g** porous PCL-TCP film (FN coating)

Next, we tried to increase cell adhesion and proliferation by coating with cell adhesive protein, fibronectin, and observe the cell behaviors by using live/dead assay as shown Fig. 8. The employed film samples are Control (Tissue culture plate), PCL film (FN coating), PCL-gelatin film, PCL-TCP film (FN coating), porous PCL film (FN coating), porous PCL-gelatin film, and porous PCL-TCP film (FN coating) (Fig. 8a, b, c, d). Significant large number of cells were observed on fibronectin-coated PCL films (Fig. 8b) surface compared to that of control polystyrene tissue culture flask (Fig. 8a). As observed less cell numbers in the porous films than the non-porous film surface in Fig. 6, the flat smooth surface (Fig. 8b) showed higher cell numbers than the porous PCL-gelatin and fibronectin-coated PCL-TCP film surface (Fig. 8c, d). This result may be from the difference in initially adhered cell number due to large pore surface diameter (approximately 100–200 μm) to that of the cells (approximately 6–10 μm) (Fig. 8e, f, g). From the result of live/dead assay, dead cells were not observed in most groups. There was no significant difference in cell morphology between the non-porous film groups, but it showed slightly directional growth behavior. Dead cells (arrow), were observed intermittently, but most of the cells were alive. Particularly, three dimensional propagation of cells along the pore structure was observed (Fig. 8g).

**Fig. 9:**
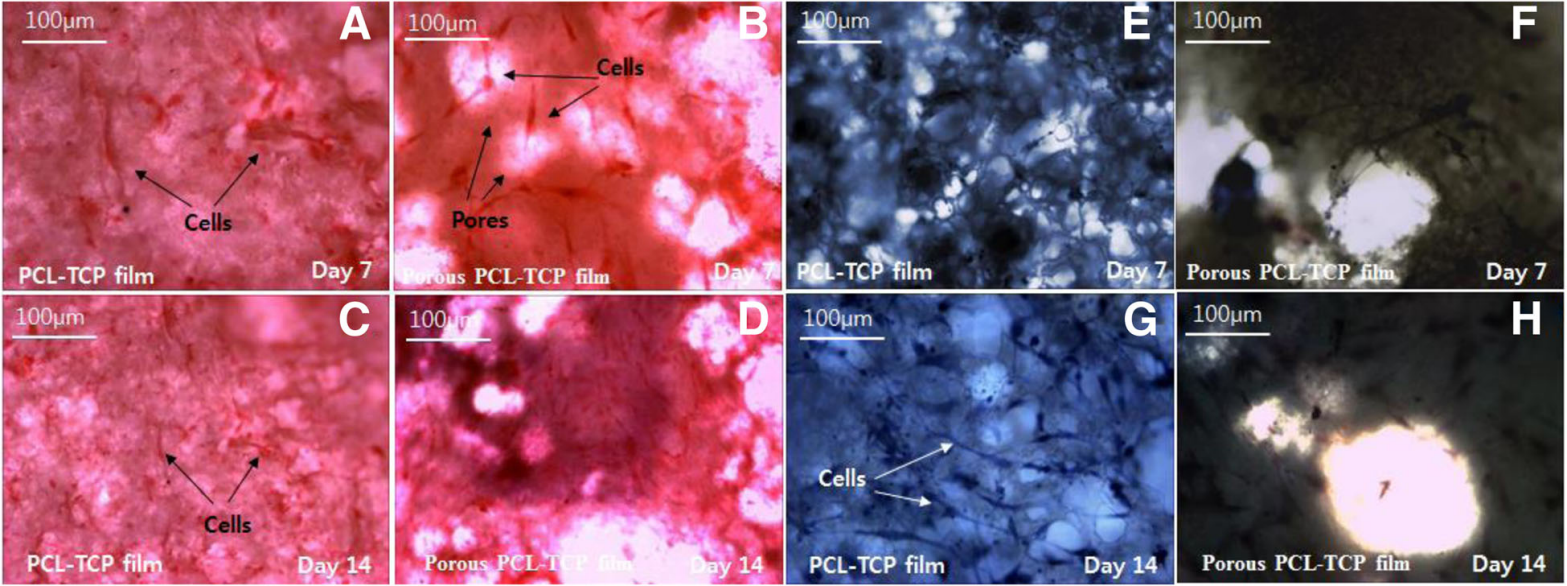
Histological analysis of PCL-TCP films with (**a**, **c**, **e**, **g**) and without (**b**, **d**, **f**, **h**) pores cultured for 7 days (**a**, **b**, **e**, **f**) and 14 days (**c**, **d**, **g**, **h**). H&E (**a**, **b**, **d**) and MT (**e**, **f**, **g**, **h**) stains results

To observe tissue regeneration on these films we stained with H&E and MT for the PCL-TCP films without (Fig. 9a, c, e, g) and with (Fig. 9b, d, f, h) pores cultured for 7 days (Fig. 9a, b, e, f) and 14 days (Fig. 9c, d, g, h). H&E stain results (Fig. 9a, b, c, d) showed more spreading of cells and secretion of extracellular matrix not only on surface but also in 3-dimensional porous structures. More cells and better spreading were observed in MT stains (Fig. 7e, g) than porous PCL-TCP films (Fig. 7f, h) at both 7 days and 14 days. These observations support previous cell viability and cell number changes, which demonstrated higher number of cells on the flat smooth surfaces of the films.

## Discussion

Diverse thin hybrid films of PCL films, PCL-TCP particles films, PCL-gelatin films and PCL-TCP particles-gelatin films were synthesized with and without pores. The TCP particles have less than 10 μm in diameter and the film thickness were in the range of approximately 40–54 μm, While the overall surface of the films was smooth without any large pores, the porous films have pore sizes ranging approximately 100 to 200 μm in with smaller pores of approximately 10 μm. TCP particles were homogeneously distributed on the surface of the films, and the analyses of the chemical structures of porous PCL films showed all the components such as PCL and TCP. Different hybrid films degraded in SBF and DMEM depending on their compositions and superficial surface areas when observed for up to 50 days. The phenomena indicated that there were biodegradation of biodegradable PCL films and TCP particles from the hybrid films. Hybrid porous structures with cell adhesion factors of gelatins show higher degradation in vitro; however, these hybrid film scaffolds have the ability to match the loss by increased cell adhesion and proliferation on to the scaffolds. When we have done in vitro culture of mesenchymal stem cells on all these films such as the films of PCL, porous PCL, PCL-TCP and porous PCL-TCP, PCL-gelatin and porous PCL-gelatin by comparing with that of control polystyrene culture flask, non-porous three groups (PCL, PCL-TCP, PCL-gelatin) show lower rate of cell proliferation rates than control. However, coating with fibronectin increased significantly their cellular interaction and proliferation as well as extracellular matrix formation, with no cell death both on the surface and inside the pores of the hybrid films. Fibronectin, a high-molecular weight (~ 440 kDa) glycoprotein of the extracellular matrix, has been known to bind to membrane-spanning receptor proteins, which is called as integrins. It plays a major role in adhesion, growth, migration and differentiation of cells for wound healing, including bone tissue regeneration [38, 39].

## Conclusion

Thin hybrid films of PCL-TCP particles were fabricated by simply solvent casting methods, and their morphologies showed homogeneous distribution of TCP particles in biodegradable PCL matrix as confirmed by EDX, light microscopy and SEM. Pores were obtained by the processing of mixing ammonium bicarbonate particles and their gas forming. The thicknesses and pores sizes of the hybrid films with and without pores were in the range of approximately 40–54 μm, and 100–200 μm, respectively, and their biodegradations were dependent on the existence of pores, showing that higher superficial films lead to quicker degradation in SBF and DMEM buffers. The hybrid films showed their own characteristics of chemical compositions and existence of calcium and demonstrated overall higher human mesenchymal stem cell adhesion, proliferation and tissue regeneration on the non-porous thin films of PCL-TCP, PCL-TCP-gelatin and PCL-TCP-fibronectin. All cells were viable and their proliferations were comparable to that of polystyrene dish. Inclusion of collagen in the PCL-TCP hybrid films induced increase in cell adhesion and proliferation, and coating of fibronectin induced remarkable in induction of cell adhesion and proliferation as well as showed high expression of extracellular matrix as observed by H&E and MT stain, which are suitable for tissue engineering applications. Thus control of hybrid film properties such as compositions of PCL and TCP particles, particle distribution, film thickness, porosity, biodegradation and employment of cell adhesive proteins could be the option for better design of tissue engineering scaffolds.
